# The Level of Knowledge, Attitude, Practice, and Adherence of Obstetricians and Gynecologists Towards the Triple Elimination Program (HIV, Syphilis, Hepatitis B) in Maternal Services at Government and Private Hospitals in Jakarta

**DOI:** 10.3390/ijerph23081090

**Published:** 2026-08-21

**Authors:** Yudianto Budi Saroyo, Baihaki, Mohammad Adya F. Dilmy, Lisa Novianti, Thalia Amila Elsiyana

**Affiliations:** Department of Obstetrics and Gynecology, Faculty of Medicine, Universitas Indonesia, Jakarta 10430, Indonesia; rickybaihaki27@gmail.com (B.); dido.dilmy@gmail.com (M.A.F.D.); lisa.novianti02@ui.ac.id (L.N.); thaliaamilae@gmail.com (T.A.E.)

**Keywords:** HIV, hepatitis B, syphilis, triple elimination, maternal services, hospital

## Abstract

**Highlights:**

**Public health relevance—How does this work relate to a public health issue?**

- Mother-to-child transmission (MTCT) of HIV, Syphilis, and Hepatitis B is a major public health challenge that causes high morbidity and mortality, especially in developing nations.
- The Triple Elimination Program is a vital public health intervention that relies heavily on the correct implementation by frontline maternal healthcare providers, such as obstetricians and gynecologists.

**Public health significance—Why is this work of significance to public health?**

- This study evaluates the actual readiness and adherence of maternal healthcare specialists in Jakarta towards implementing the Triple Elimination Program.
- This study exposes significant operational gaps, revealing that approximately half of the surveyed specialists lack adequate knowledge, attitudes, or practical application regarding the program’s guidelines.

**Public health implications—What are the key implications or messages for practitioners, policy makers and/or researchers in public health?**

- Hospital administrators and policy makers must urgently implement targeted educational and training interventions to improve providers’ understanding and attitudes toward the Triple Elimination guidelines.
- Continuous monitoring and systemic support are needed across government and private hospitals to ensure strict compliance and effectively reduce MTCT rates.

**Abstract:**

Background: Human Immunodeficiency Virus (HIV), Syphilis, and Hepatitis B significantly contribute to high morbidity and mortality rates associated with mother-to-child transmission (MTCT), particularly in developing nations. The study delves into the adherence levels of obstetricians and gynecologists to the Triple Elimination Program in Jakarta. Methods: This study employed a descriptive cross-sectional design, conducted across government and private hospitals in Jakarta from January to December 2023. The study sample comprises obstetricians and gynecologists selected through consecutive sampling. Data collection involves questionnaires measuring knowledge, attitude, practice, and adherence, with validity and reliability assessed through pilot studies. Results: Out of the 106 respondents, 47.2% lacked adequate knowledge of the Triple Elimination Program, while 52.8% demonstrated higher knowledge. Insufficient attitude was observed in 50% of respondents, with the remaining 50% reporting a good attitude towards the program. Inadequate practice was noted in 49% of respondents and 51% perceived themselves as having fair practice. Most respondents demonstrated only fair adherence (74.5%), whereas only 25.5% achieved good adherence. Conclusions: The research findings highlight existing gaps in knowledge, attitude, practice, and adherence among healthcare providers, emphasizing the need for improvement in these areas.

## 1. Introduction

Human Immunodeficiency Virus (HIV), Syphilis, and Hepatitis B are diseases that contribute to high morbidity and mortality rates in mother-to-child transmission (MTCT), particularly in developing countries [1,2]. The strategy for the elimination of MTCT (EMTCT) was initially launched by the World Health Organization (WHO) in 2014, primarily targeting HIV and Syphilis, followed by Hepatitis B.

Various studies have reported the status and achievements of triple elimination in several countries. Visser et al., evaluating birth data in the Netherlands from 2009 to 2015, reported that the coverage of triple elimination screening had exceeded >99% for each year, with a low prevalence of infections: HIV 0.06%, Syphilis 0.06%, and HBV 0.29% [3]. Woodring et al. assessed the achievement of EMTCT for HBV in the Western Pacific region and found an increase in Hepatitis B vaccination coverage at birth from 63% to 85% during the period 2005–2017. This contributed to a decrease in chronic hepatitis infections in children to <1% in 25 (69%) countries in the region [4,5]. In a study by Zhang et al. in Cambodia, a developing country in the same region as Indonesia, integrating triple elimination into service standards for 370,000 pregnant women could result in overall health cost reduction of up to USD 380,000 or approximately IDR 5.6 billion [6,7]. In Jakarta, recent surveillance has shown that despite the rollout of EMTCT policies, coverage of HIV, syphilis, and hepatitis B testing in antenatal care remains suboptimal, and mother-to-child transmission of these infections persists, particularly among women attending private facilities and those with lower socioeconomic status [8,9].

The Indonesian government integrated HIV, Syphilis, and Hepatitis B services through the Triple Elimination Program in 2017, regulated by the Minister of Health Regulation of the Republic of Indonesia Number 52 of 2017 concerning the Elimination of HIV, Syphilis, and Hepatitis B Transmission from Mother to Child and Guidelines for the Elimination of HIV, Syphilis, and Hepatitis B Transmission from Mother to Child in 2017 [10,11]. Through this program, the government-initiated strategies to support the success of triple elimination, including improving access to and quality of health services for mothers and children according to standards, enhancing the role of health service facilities in necessary management, and increasing resources in the health sector.

According to the Ministry of Health of Indonesia, as of June 2014, the EMTCT policy in Indonesia had not been well implemented. For example, only 2.5% of pregnant women underwent HIV testing [12]. Health facility systems that are not supportive, lack of skilled human resources, personnel turnover, budget constraints, and lack of awareness and knowledge among pregnant women are the main obstacles hindering access to mother-to-child transmission prevention services [13]. Adherence barriers to clinical guidelines may be related to individual patients, service providers, individual health, individual group, organizational context, social and cultural context of the healthcare system [14,15,16]. Adherence with clinical guidelines will enhance the healthcare delivery process [17,18,19].

In Indonesia, there is currently no research evaluating the level of adherence of healthcare professionals, especially obstetricians and gynecologists, in the implementation of triple elimination screening. In Jakarta, in 2022, there were 655 obstetricians and gynecologists [20]. Adherence of obstetricians and gynecologists with triple elimination can serve as a crucial indicator to break the chain of transmission from mother to child and provide an overview of the implementation of triple elimination screening in healthcare facilities, especially in DKI Jakarta. Therefore, research on adherence with triple elimination, especially in maternal services in hospitals, particularly in Jakarta, is highly necessary [21,22,23,24]. The primary objective of this study was to assess the level of knowledge, attitude, practice, and adherence of obstetricians and gynecologists regarding triple elimination of mother-to-child transmission in hospitals in Jakarta. Secondary objectives were to identify specific gaps in knowledge, attitude, practice, and adherence that may affect implementation.

## 2. Materials and Methods

### 2.1. Study Design

This analytical cross-sectional study was conducted in government and private hospitals in Jakarta from January 2023 to December 2023.

### 2.2. Questionnaire Development and Validation

The questionnaire used in this study was a self-developed instrument because no previously validated questionnaire specifically assessing obstetricians’ and gynecologists’ knowledge, attitudes, practices, and adherence regarding the implementation of the Indonesian Triple Elimination Program was available (Table 1). The questionnaire was developed based on the Indonesian Ministry of Health Regulation No. 52/2017, the National Guideline for Triple Elimination (2019), WHO recommendations, and relevant published literature. Prior to data collection, the questionnaire underwent expert review, validity testing, and reliability testing. The administered instrument contained 16 knowledge items, 9 attitude items, 12 practice items, and 2 adherence items. Item-total Pearson correlations from a 30-participant pilot were used only as item-level internal-structure screening and were not interpreted as full construct validation. All 16 knowledge items and both adherence items were retained. Attitude item 8 and practice items 2, 3, 4, 8, and 12 were excluded from domain scoring because they did not meet the prespecified item-correlation criterion. Consequently, the analysed scores comprised 16 knowledge, 8 attitude, 7 practice, and 2 adherence items.

The knowledge domain employed a three-level scoring system in which correct responses were scored as 2, unsure responses as 1, and incorrect responses as 0, yielding a total score ranging from 0 to 40. Attitude and practice items were assessed using a five-point Likert scale, while adherence items were scored using the predefined ordinal response scale. Attitude items used a five-point agreement scale (1 = strongly disagree to 5 = strongly agree), whereas practice items used a five-point frequency scale (1 = never to 5 = always). Adherence items were coded 1 = never, 2 = sometimes, and 3 = always. Attitude item 4 and practice items 9, 10, and 11 were negatively worded and reverse-coded as 6 minus the original response before summation. The adherence domain consisted of two indicator items representing two essential clinical practices recommended in the Indonesian Triple Elimination guideline: (1) performing HIV, syphilis, and hepatitis B screening during the first antenatal care visit and (2) referring pregnant women with positive screening results to the appropriate specialist. Because these items were designed to assess compliance with key clinical recommendations rather than to measure a multidimensional latent construct, the adherence domain was treated as an indicator-based measure instead of a comprehensive psychometric adherence indicator.

Respondents were classified into good and fair categories using the mean score of each domain as the cut-off value. Since no validated cut-off values currently exist for this newly developed instrument, the mean score was used as an exploratory cut-off to categorize respondents into the ‘good’ and ‘fair’ groups. Respondents with scores equal to or greater than the domain mean were categorized as good, whereas those scoring below the mean were categorized as fair. The mean cut-off values were 30.97 for knowledge, 34.73 for attitude, 22.23 for practice, and 5.70 for adherence. Construct validity was evaluated using the Pearson Product Moment correlation test, whereas internal consistency reliability was assessed using Cronbach’s alpha coefficient.

### 2.3. Study Population and Sampling

The study population consisted of obstetricians and gynecologists practicing in government and private hospitals in Jakarta. Respondents were recruited from selected hospitals that agreed to participate in the study. Eligible respondents were obstetricians and gynecologists who were actively practicing during the study period, fulfilled the study eligibility criteria, and were willing to participate. Physicians who declined participation or submitted incomplete questionnaires were excluded from the study. The minimum required sample size was calculated using the Lemeshow formula for estimating a population proportion with a 95% confidence level, an assumed proportion of 50%, and an absolute precision of 10%. The minimum sample size was 96 respondents. To compensate for potential non-response or incomplete questionnaires, an additional 10% was added, resulting in a target sample size of 106 respondents. Respondents were recruited using a consecutive sampling technique. All eligible physicians encountered during the study period at the participating hospitals were consecutively invited until the predetermined sample size of 106 respondents was achieved. Before enrollment, all eligible physicians received a detailed explanation of the study objectives and procedures, and written informed consent was obtained. During the recruitment period, 106 eligible physicians were invited to participate, and all agreed to participate and completed the questionnaire. No respondent declined participation, withdrew from the study, or submitted incomplete questionnaires. Therefore, all 106 respondents were included in the final analysis, resulting in a 100% response rate among invited respondents. The study was conducted in accordance with the principles of the Declaration of Helsinki and was approved by the Research Ethics Committee of the Faculty of Medicine, Universitas Indonesia–Cipto Mangunkusumo Hospital (Protocol No. 23-01-0049). Written informed consent was obtained from all respondents before data collection.

### 2.4. Variables

This study is descriptive in nature, thus not involving identifiable independent, dependent, and confounding variables. Knowledge variables were measured using a categorical true/false scale. Attitude and practice variables were measured using a Likert scale. Adherence was measured using an ordinal scale with options of never, sometimes, and always. All variables were measured using a questionnaire.

For the variables under study, respondents’ average values will be categorized into five categories: very high, high, moderate, low, and very low. The highest possible value in this study is 5, while the lowest is 1. The category class intervals obtained are as follows: Very high (4.24–5.00), high (3.43–4.23), moderate (2.62–3.42), low (1.81–2.61), and very low (1.00–1.80).

### 2.5. Quality Assurance

The questionnaire underwent validity and reliability tests initially through a pilot study involving 30 hospital staff. Instrument validity in this research was assessed using Pearson Product Moment analysis. Pearson item-total correlations were used to screen individual items against the stated r-table threshold, and Cronbach’s alpha was used to describe internal consistency. These procedures support item-level screening and internal consistency only; they do not constitute comprehensive construct validation. The conceptual domains were retained as knowledge (factual information), attitude (evaluative beliefs), practice (self-reported clinical behaviour), and adherence (two guideline-linked clinical indicators). Some attitude items also contain clinical-management content, which is acknowledged as a limitation of domain specificity. The instrument is deemed valid if the calculated r value exceeds the tabulated r value. Subsequently, the questionnaire underwent reliability testing using Cronbach’s Alpha analysis, where if a variable shows more than 0.70, it can be considered reliable or consistent in measurement.

### 2.6. Data Management and Analysis

Descriptive statistics were used to summarize respondents’ demographic characteristics and questionnaire scores. Bivariate analyses were subsequently performed to examine the associations between knowledge, attitude, and practice with physicians’ adherence to the Triple Elimination Program using the Chi-square test. Odds ratios (ORs) with 95% confidence intervals (95% CIs) were also calculated. To identify independent predictors of adherence, variables included in the conceptual framework (knowledge, attitude, and practice) were entered simultaneously into a binary logistic regression model using the enter method. Statistical significance was defined as a two-sided *p*-value of <0.05. All statistical analyses were performed using IBM SPSS Statistics version 26.0.

## 3. Results

### 3.1. Socio-Demographic Characteristics of Respondents

The study was conducted in December 2023 by distributing questionnaires to 106 respondents. All eligible physicians who were approached agreed to participate and completed the questionnaire. No questionnaires were excluded because of incomplete responses. Based on Table 2, it is known that out of 106 respondents, females comprised a larger proportion compared to males, with 60.4% and 39.6% respectively. Most respondents were in the age group of 31–40 years, constituting 71 respondents (67%), and 27 respondents (25.5%) were in the age group of 41–50 years. Out of 106 respondents, the majority worked in Private Hospitals (50%), and in Government Hospitals (17.9%). Furthermore, based on work experience, the highest proportion was in the >6 months–5 years category, with 55 respondents (51.9%), and 6–10 years with 34 respondents (32.1%).

Based on Table 3, 50 respondents (47.2%) had inadequate knowledge, and with 56 respondents (52.8%) having good knowledge, 53 respondents (50.0%) had an inadequate attitude. Fair practice was observed in 54 respondents (51.0%), slightly exceeding the number with good practice. Additionally, there was an incredibly high number of respondents with fair adherence (74.5%).

### 3.2. Knowledge

Although 56 respondents (52.8%) were categorized as having higher knowledge, item-level analysis identified several clinically important knowledge gaps. The highest incorrect response rate was observed for the risk of mother-to-child transmission of hepatitis B without prophylaxis (24.5%), followed by early infant HIV diagnosis using DNA PCR at six weeks (15.1%) and the recommended follow-up schedule for infants born to HBsAg-positive mothers (12.3%). These findings indicate that important gaps remain in specific clinical aspects of the Triple Elimination Program despite the overall knowledge level. Table 4 presents the item-level distribution of respondents’ knowledge.

### 3.3. Attitude

Table 5 presents attitude towards triple elimination. Respondents with low index scores responded negatively towards the following statements: every pregnant woman who is HIV/Syphilis/Hepatitis B positive must be provided with standard services including therapy, delivery assistance, breastfeeding counseling, and contraception; management for pregnant women with HIV infection is treated with fixed-dose combination ARVs, 1 × 1 dose per day for life; management for pregnant women with positive Syphilis results is Benzathine Penicillin G 2.4 million IU IM single dose in early stage, repeated twice with a one-week interval or referred; and I explain about triple elimination when asked by pregnant women. Responses to each attitude and practice questionnaire item are presented individually. Each row in Table 5 and Table 6 represents a single questionnaire item, and all frequencies are reported as the number and percentage of respondents based on the total sample of 106 respondents.

### 3.4. Practice

Respondents with low index scores responded negatively towards the following statements: I only perform HIV testing on pregnant women with risk factors and symptoms; I only perform Syphilis testing on pregnant women with risk factors and symptoms; I only perform Hepatitis B testing on pregnant women with risk factors and symptoms; I conduct triple elimination screening even if pregnant women do not request it. Answers with low index scores but negative statements, where responding disagree is considered good response, indicating Respondents’ Practice is already sufficiently Positive in supporting the Triple Elimination Program. Table 6 presents practice towards triple elimination.

### 3.5. Adherence

Low index score responses were observed in the statement “I request screening for HIV, Syphilis, and Hepatitis B to be performed on all pregnant women at the first antenatal visit to the Hospital”, where 22 individuals (20%) answered sometimes. Table 7 presents adherence towards Triple Elimination Program.

### 3.6. Factors Associated with Adherence to the Triple Elimination Program

Bivariate analyses demonstrated that none of the investigated variables were significantly associated with physicians’ adherence to the Triple Elimination Program. Knowledge was not significantly associated with adherence (χ^2^ = 0.319, *p* = 0.572; OR = 1.286, 95% CI: 0.536–3.086). Similarly, attitude (χ^2^ = 0.050, *p* = 0.824; OR = 1.105, 95% CI: 0.461–2.648) and practice (χ^2^ = 2.094, *p* = 0.148; OR = 1.930, 95% CI: 0.787–4.734) showed no statistically significant association with adherence (Table 8).

Variables considered theoretically relevant (knowledge, attitude, and practice) were subsequently included in a binary logistic regression model. The overall model was not statistically significant (Omnibus test χ^2^ = 2.280, df = 3, *p* = 0.516), indicating that the model did not significantly improve prediction of adherence compared with the null model. The model explained only 2.1% of the variance according to the Cox and Snell R^2^ and 3.1% according to the Nagelkerke R^2^. None of the independent variables remained significant predictors of adherence after adjustment, including knowledge (adjusted OR = 1.174, *p* = 0.735), attitude (adjusted OR = 0.862, *p* = 0.761), and practice (adjusted OR = 1.953, *p* = 0.167) (Table 9).

Additional bivariate analyses were performed to examine the associations between respondents’ characteristics (gender, age, work experience, and hospital type), KAPA domains, and adherence. No statistically significant associations were identified (all *p* > 0.05).

## 4. Discussion

The findings indicate that nearly half of the respondents had insufficient knowledge, with 50 individuals (47.2%) lacking knowledge, while 56 respondents demonstrated good knowledge [25,26]. Item-level analysis revealed several clinically important knowledge gaps despite the overall proportion of respondents with higher knowledge. The highest incorrect response rate was observed for the risk of mother-to-child transmission of hepatitis B without prophylaxis (24.5%), followed by early infant HIV diagnosis using DNA PCR at six weeks (15.1%) and the recommended follow-up schedule for infants born to HBsAg-positive mothers (12.3%). These findings suggest that while physicians generally understand the Triple Elimination Program, important gaps remain in specific clinical-management recommendations. Such deficiencies may affect timely diagnosis, neonatal follow-up, and prevention of mother-to-child transmission. Therefore, continuing medical education should place greater emphasis on hepatitis B prevention, early infant HIV diagnostic protocols, and follow-up recommendations for exposed infants. In Indonesia, acceptance of HIV testing among pregnant women has been shown to depend strongly on recommendations from health providers, knowledge of HIV transmission, and husband or family support. Limited explanation from health workers and insufficient knowledge may reduce willingness to undergo testing, thereby hindering early diagnosis and timely treatment [27]. Reasons for pregnant women not undergoing HIV testing include limited knowledge about its importance, counseling services, confidentiality, communication issues, and beliefs about risk practice, thus hampering the Triple Elimination program [28].

Regarding attitude, 53 individuals (50%) exhibit insufficient attitude, while 53 (50%) perceive themselves as having a good attitude. Activities of the Triple Elimination Program involve promotion, surveillance, early detection, and case management, with healthcare workers playing a vital role. Healthcare providers’ attitude influences pregnant women’s utilization of triple elimination services, aligning with Green’s theory. Attitudes are influenced by knowledge, impacting healthcare providers’ practice in deciding whether to utilize these services [10].

The finding that nearly half of respondents had insufficient knowledge is consistent with reports from other Indonesian and Southeast Asian settings where provider knowledge remains uneven despite EMTCT policy implementation. Variations in training exposure, guideline dissemination, and institutional support may explain these differences. The limited knowledge observed in this study may reflect insufficient continuing medical education, variable dissemination of updated guidelines, and differences in hospital policies between public and private facilities. In addition, providers in private hospitals may experience different implementation priorities, access to training, or supervision compared with those in government hospitals, which may contribute to uneven knowledge of triple elimination procedures [29].

The finding that a substantial proportion of respondents demonstrated inadequate knowledge and generally positive attitudes, yet adherence remained mostly fair rather than consistently good, suggests a knowing-doing gap in triple elimination implementation. This pattern indicates that improving adherence requires more than knowledge transfer alone. In addition to continuing medical education, implementation strategies such as audit-and-feedback, reminder systems, clinical champions, standardized screening protocols, and stronger institutional accountability may be necessary to translate provider knowledge and attitude into routine screening practice [9]. Evidence from recent intervention studies suggests that targeted training, audit-and-feedback, and integration of EMTCT guidelines into routine obstetric workflows can significantly increase provider adherence to HIV, syphilis, and hepatitis B screening and management in antenatal care. Implementing structured continuing medical education and supportive supervision for obstetricians and gynecologists in Jakarta could help translate knowledge and attitudes into consistent practice [30].

Inadequate practice persists, with 54 individuals (51%) lacking, while 52 (49%) perceive themselves as behaving well. Healthcare provider support, especially in advising triple elimination examinations, is crucial, aligning with L. Green’s theory and emphasizing the role of healthcare providers in encouraging testing. Additionally, the research underscores the importance of reinforcement in changing practice, especially when individuals possess potential like higher knowledge and attitude. Integrated service-delivery models, such as one-stop antenatal clinics where HIV, syphilis, and hepatitis B testing are offered routinely with point-of-care diagnostics, have been associated with higher screening coverage and reduced MTCT rates in comparable settings. Strengthening electronic medical records, standardized screening algorithms, and task-shifting to midwives or nurses for initial testing could improve implementation of triple elimination in Jakarta hospitals [30]. On the other hand, unsupportive practice from healthcare providers, particularly doctors, can undermine the importance of Triple Elimination examinations, impacting maternal and child health [31]. Most respondents demonstrated only fair adherence (74.5%) and good adherence reaching up to 25.5%. Mandating HIV testing presents dilemmas, with healthcare providers’ role crucial in encouraging pregnant women to undergo testing. A considerable proportion of pregnant women forego HIV testing due to insufficient information from healthcare providers, highlighting the significant influence of provider–patient relationships on testing decisions [26,32]. To further investigate factors associated with adherence, additional inferential statistical analyses were performed. Bivariate analyses demonstrated that knowledge, attitude, and practice were not significantly associated with physicians’ adherence to the Triple Elimination Program. Although respondents with good practice tended to demonstrate higher adherence than those with poor practice, this association did not reach statistical significance. Likewise, knowledge and attitude were not significantly associated with adherence. These findings suggest that improvements in knowledge, attitude, or self-reported practice alone may not necessarily translate into better adherence to the Triple Elimination Program. The multivariable logistic regression analysis further confirmed these findings. After adjustment for knowledge, attitude, and practice, none of these variables were identified as independent predictors of adherence. The overall regression model was also not statistically significant. This lack of statistically significant predictors may be attributed to several methodological constraints, including the relatively small sample size, limited statistical power, and the small number of respondents classified as having good adherence. Beyond these limitations, the findings indicate that physicians’ adherence is likely influenced by factors outside of individual knowledge, attitudes, and practices.

However, the confidence intervals were wide, the lower adjusted confidence limit was close to 1, and the overall multivariable model was not significant. The association is therefore exploratory and imprecise, and the cross-sectional design does not establish directionality or causation.

Organizational support, institutional policies, implementation of standardized clinical protocols, availability of diagnostic facilities, workload, continuing professional education, supportive supervision, and differences in healthcare financing under Indonesia’s National Health Insurance (JKN) system may all contribute to adherence behaviour. These organizational and health system factors were not evaluated in the present study but may explain why acceptable levels of knowledge, attitude, and practice did not consistently translate into optimal adherence. Future multicentre studies incorporating these contextual determinants are therefore recommended to better identify predictors of physicians’ adherence to the Triple Elimination Program. The adherence findings should be interpreted in the context of the study instrument. The adherence domain was intentionally limited to two key clinical indicators that represent essential physician behaviors within the Triple Elimination Program, namely universal screening at the first antenatal care visit and appropriate referral of women with positive screening results. Therefore, the findings reflect physicians’ adherence to these critical program recommendations rather than a comprehensive assessment of all components of Triple Elimination implementation. Future studies should develop and validate a more comprehensive adherence instrument encompassing additional dimensions such as documentation, treatment initiation, follow-up, case notification, and continuity of care.

Community-based models that combine antenatal care with partner testing, peer support, and digital health messaging have shown promise in increasing uptake of HIV and syphilis testing and improving linkage to care. Adapting such models—for example, mobile phone reminders, social media campaigns, and collaboration with community health workers—could enhance pregnant women’s engagement with triple elimination services in Jakarta and reduce residual MTCT risk. The absence of significant associations between adherence and respondents’ characteristics suggests that implementation challenges may be shared across different physician groups, indicating the need for system-wide interventions rather than subgroup-specific strategies.

Hospital policies, financing mechanisms, training exposure, diagnostic availability, workload, and supervision may influence implementation. However, these factors were not measured in this study. Future multicentre studies should directly evaluate their effects using hospital-level identifiers and objective screening records to better understand variations in implementation across healthcare settings.

## 5. Limitations

This study has several limitations that should be considered when interpreting the findings. The study was conducted only in Jakarta, which may limit the generalizability of the results to obstetricians and gynecologists practicing in other regions of Indonesia where healthcare resources, institutional policies, and implementation of the Triple Elimination Program may differ. In addition, the cross-sectional design provides only a snapshot of physicians’ knowledge, attitude, practice, and adherence at a single point in time, precluding the assessment of temporal changes or causal relationships among these variables.

Respondents were recruited using a consecutive sampling technique from selected participating hospitals until the predetermined sample size was achieved. Although all eligible physicians who were invited agreed to participate and completed the questionnaire, the use of non-probability sampling may have introduced selection bias and limited the representativeness of the study population. Therefore, caution should be exercised when extrapolating these findings to all obstetricians and gynecologists practicing in Jakarta or other regions of Indonesia.

Furthermore, adherence and practice were assessed using self-administered questionnaires, which are inherently respondent to recall bias and social desirability bias and may not fully reflect actual clinical practice. The study also relied exclusively on self-reported data without direct observation of clinical practice or verification using hospital medical records or screening databases. The adherence domain included two key indicator items and should be interpreted as a measure of compliance with selected Triple Elimination practices rather than overall adherence. Future studies should employ a more comprehensive adherence instrument. Future multicenter studies employing probability sampling and objective measures of clinical practice are recommended to improve the representativeness, validity, and generalizability of the findings.

## 6. Conclusions

Knowledge of the Triple Elimination Program among healthcare providers remained insufficient in a substantial proportion of respondents, while attitude, practice, and adherence were generally moderate. These findings indicate the need for targeted continuing medical education, stronger hospital-level screening policies, routine supervision, and clearer accountability mechanisms to improve provider adherence and strengthen triple elimination implementation in hospital settings.

## Figures and Tables

**Table 1:** Characteristics, Scoring System, Validity, Reliability, and Classification of the Self-Developed Questionnaire.

Domain	Objective	No. of Items	Valid Items	Response Scale	Scoring Procedure	Possible Score	Mean Score (Cut-Off)	Classification	Pearson’s r	Cronbach’s α
**Knowledge**	To assess obstetricians’ and gynecologists’ knowledge regarding the Triple Elimination Program, including HIV, syphilis, hepatitis B, screening, diagnosis, treatment, and national recommendations.	20	20	Correct/ Unsure/ Incorrect	Correct = 2; Unsure = 1; Incorrect = 0	0–40	30.97	Good ≥ 30.97; Fair < 30.97	0.561–0.635	0.715
**Attitude**	To assess respondents’ attitudes toward the implementation of the Triple Elimination Program during antenatal care.	9	9	Five-point Likert scale (1 = Strongly disagree to 5 = Strongly agree)	Scores of 1–5 for each item	9–45	34.73	Good ≥ 34.73; Fair < 34.73	0.520–0.815	0.799
**Practice**	To assess routine clinical practices related to Triple Elimination in diagnosis, counselling, and management.	12	12	Five-point Likert scale (1 = Never to 5 = Always)	Scores of 1–5 for each item	12–60	22.23	Good ≥ 22.23; Fair < 22.23	0.365–0.778	0.861
**Adherence**	To assess physicians’ adherence to the Indonesian National Guideline for Triple Elimination.	2	2	Three-point ordinal scale (1 = Never, 2 = Sometimes, 3 = Always)	Scores of 1–3 for each item	2–6	5.70	Good ≥ 5.70; Fair < 5.70	0.583–0.778	0.819

**Note:** Respondents were classified as having good or fair knowledge, attitude, practice, and adherence using the mean score of each domain as the cut-off value.

**Table 2:** Socio-demographic characteristics of respondents.

Characteristics	N (106)	Percentage
Gender		
Male	42	39.6%
Female	64	60.4%
Age (years)		
<30	1	0.9%
>50	7	6.6%
31–40	71	67.0%
41–50	27	25.5%
Work Experience		
>6 months–5 years	55	51.9%
>20 years	6	5.7%
11–15 years	7	6.6%
16–20 years	4	3.8%
6–10 years	34	32.1%
Hospital Type		
Government	19	17.9%
Government & Private	34	32.1%
Private	53	50.0%
Total	106	100.0%

**Table 3:** Distribution of knowledge, attitude, practice, and adherence.

Variable	Good, *n* (%)	Fair *n* (%)	Mean (SD)	Observed Range
Knowledge	56 (52.8)	50 (47.2)	30.97 (1.46)	26–32
Attitude	53 (50.0)	53 (50.0)	35.15 (3.58)	12–40
Practice	52 (49.0)	54 (51.0)	28.84 (4.25)	19–35
Adherence	27 (25.5)	79 (74.5)	5.70 (0.55)	4–6

**Table 4:** Knowledge of triple elimination.

No.	Statements	False	True
1	Human Immunodeficiency Virus (HIV) is a virus that attacks the immune system and if left untreated can weaken human immunity leading to Acquired Immune Deficiency Syndrome (AIDS).	0	106
2	Without ART medication, the risk of HIV transmission from mother to child is 20–45%.	7	99
3	HIV infection is diagnosed through qualitative DNA PCR testing in infants aged 6 weeks or older, and they are declared HIV-infected if the test result is positive.	16	90
4	Syphilis is one type of sexually transmitted infection caused by the bacterium Treponema Pallidum.	0	106
5	Without treatment, the risk of syphilis transmission from mother to child is 69–80%.	9	97
6	Syphilis infection is determined through Plasma Reagin test (RPR) titers examination in infants at 3 months of age and in the mother, and a baby is diagnosed with syphilis if the baby’s titer is more than 4 times higher than the mother’s titer. For example, if the mother’s titer is 1:4, then the baby’s titer should be 1:16 or higher than 1:32.	8	98
7	Hepatitis B is a contagious disease characterized by inflammation of the liver caused by the Hepatitis B virus.	1	105
8	Without treatment, the risk of hepatitis B transmission from mother to child is over 90%.	26	80
9	Hepatitis B infection is determined through HBsAg testing when the baby is aged 9 months or older, and they are diagnosed with Hepatitis B if the HBsAg test is positive.	13	93
10	Triple elimination refers to the reduction of the risk of HIV, Syphilis, and Hepatitis B transmission from mother to child.	8	98
11	HIV, Syphilis, and Hepatitis B infections are diseases that can be transmitted from an infected mother to her child during pregnancy, childbirth, and breastfeeding.	5	101
12	Efforts to eliminate HIV, Syphilis, and Hepatitis B transmission are carried out collectively because these infections have relatively similar transmission patterns, such as through sexual intercourse, blood exchange or contamination, and vertically from mother to child.	2	104
13	Early detection of triple elimination is achieved through blood tests on pregnant women at least once during pregnancy.	2	104
14	Management of triple elimination cases is indicated for every infected pregnant woman until breastfeeding.	6	100
15	Management for babies born to mothers infected with HIV, Syphilis, and/or Hepatitis B includes immunization, prophylaxis, early detection, or treatment according to the provisions of the law and regulations.	2	104
16	There are national guidelines for the prevention of triple elimination transmission.	4	102

**Table 5:** Distribution of respondents’ answers to each attitude questionnaire item.

No.	Statements	Response (*n* = 106)					Total	Avg	Cat
		SA (*n*%)	A (*n*%)	N (*n*%)	D (*n*%)	SD (*n*%)			
1	Every pregnant woman must receive information regarding triple elimination.	42 (39.6)	64 (60.4)	0 (0.0)	0 (0.0)	0 (0.0)	106	4.4	Very High
2	Every pregnant woman is required to undergo HIV, Syphilis, and Hepatitis B testing at least once during Antenatal Care (ANC) visits.	1 (0.9)	7 (6.6)	71 (67.0)	27 (25.5)	0 (0.0)	106	2.8	Moderate
3	Doctors must provide education to pregnant women about the importance of triple elimination testing.	19 (17.9)	34 (32.1)	53 (50.0)	0 (0.0)	0 (0.0)	106	3.7	High
4	If a pregnant woman refuses HIV, syphilis, and hepatitis B screening, the physician will refuse to provide healthcare services.	55 (51.9)	6 (5.7)	7 (6.6)	4 (3.8)	34 (32.1)	106	3.4	High
5	Every pregnant woman who is HIV/Syphilis/Hepatitis B positive must be provided with standard services including therapy, delivery assistance, breastfeeding counseling, and contraception	2 (1.9)	5 (4.7)	19 (17.9	29 (27.4)	51 (48.1)	106	1.8	Low
6	Management for pregnant women with HIV infection is treated with fixed-dose combination ARVs, 1 × 1 dose per day for life	1 (0.9)	5 (4.7)	27 (25.5)	25 (23.6)	48 (45.3)	106	1.9	Low
7	Management for pregnant women with positive Syphilis results is Benzathine Penicillin G 2.4 million IU IM single dose in early stage, repeated twice with a one-week interval or referred	17 (16.0)	13 (12.3)	12 (11.3)	12 (11.3)	52 (49.1)	106	2.3	Low
8	I explain about triple elimination when asked by pregnant women.	2 (1.9)	5 (4.7)	23 (21.7)	76 (71.7)	0 0	106	2.3	Low
Total								2.8	Moderate

SA = strongly agree; A = agree; N = neutral; D = disagree; SD = strongly disagree. **Note:** Values are presented as number of respondents (*n*) and percentage (%) for each response category. Each row represents one questionnaire item.

**Table 6:** Distribution of respondents’ answers to each practice questionnaire item.

No.	Statements	Response (*n* = 106)					Total	Avg	Cat
		SA (*n*%)	A (*n*%)	N (*n*%)	D (*n*%)	SD (*n*%)			
1	For every visiting pregnant woman, I explain about triple elimination.	66 (62.3)	14 (13.2)	10 (9.4)	5 (4.7)	11 (10.4)	106	4.1	High
2	I explain about triple elimination even if pregnant women do not ask.	58 (54.7)	17 (16.0)	10 (9.4)	9 (8.5)	12 (11.3)	106	3.9	High
3	I conduct triple elimination testing if requested by pregnant women.	72 (67.9)	13 (12.3)	6 (5.7)	4 (3.8)	11 (10.4)	106	4.2	High
4	I conduct triple elimination screening even if pregnant women do not request it	2 (1.9)	7 (6.6)	97 (91.5)	0 0	0 0	106	3.1	Moderate
5	I only perform HIV testing on pregnant women with risk factors and symptoms	1 (0.9)	2 (1.9)	7 (6.6)	96 (90.6)	0 0	106	2.1	Low
6	I only perform Syphilis testing on pregnant women with risk factors and symptoms	1 (0.9)	2 (1.9)	11 (10.4)	92 (86.8)	0 0	106	2.2	Low
7	I only perform Hepatitis B testing on pregnant women with risk factors and symptoms	29 (27.4)	24 (22.6)	24 (22.6)	12 (11.3)	17 (16.0)	106	3.3	Moderate
	Total							3.3	Moderate

SA = strongly agree; A = agree; N = neutral; D = disagree; SD = strongly disagree. Note: Values are presented as number of respondents (*n*) and percentage (%) for each response category. Each row represents one questionnaire item.

**Table 7:** Adherence towards Triple Elimination Program.

No.	Statements	Response (*n* = 106)			Total	Avg
		Always	Sometimes	Never		
1	I screen for HIV, Syphilis, and Hepatitis B to be performed on all pregnant women at the first antenatal visit to the Hospital	82 (77.4)	22 (20.8)	2 (1.9)	106	2.75
2	I recommend pregnant women with positive results on HIV, syphilis, or hepatitis B screening to consult with relevant specialists.	100 (94.3)	6 (5.7)	0 0	106	2.94

**Table 8:** Association between knowledge, attitude, practice and adherence.

Variable	Poor Adherence *n* (%)	Good Adherence *n* (%)	OR (95% CI)	*p*-Value
Knowledge				
Poor	14 (51.9)	36 (45.6)	1.286 (0.536–3.086)	0.572
Good	13 (48.1)	43 (54.4)		
Attitude				
Poor	14 (51.9)	39 (49.4)	1.105 (0.461–2.648)	0.824
Good	13 (48.1)	40 (50.6)		
Practice				
Poor	17 (63.0)	37 (46.8)	1.930 (0.787–4.734)	0.148
Good	10 (37.0)	42 (53.2)		

**Table 9:** Binary logistic regression analysis of factors associated with adherence.

Variable	Adjusted OR	95% CI	*p*-Value
Knowledge	1.174	0.462–2.984	0.735
Attitude	0.862	0.331–2.243	0.761
Practice	1.953	0.756–5.047	0.167

## Data Availability

The raw data supporting the conclusions of this article will be made available by the authors on request.

## Abbreviations

The following abbreviations are used in this manuscript:

HIV	Human Immunodeficiency Virus
MTCT	Mother-to-child transmission
EMTCT	Elimination of mother-to-child transmission
WHO	World Health Organization
